# Essential elements for learning to eat: guidance to support families with infants and young children

**DOI:** 10.3389/fped.2025.1493780

**Published:** 2025-03-27

**Authors:** Wendy Sue Swanson, Erin Sundseth Ross, Luz Adriana Matiz, Laura Czerkies, Lyndsey R. Huss, Sarah Smith-Simpson, Jodi Bettler, Susan Pac

**Affiliations:** ^1^ Sean N. Parker Center for Allergy and Asthma Research, Stanford University, Palo Alto, CA, United States; ^2^ Feeding FUNdamentals LLC, Longmont, CO, United States; ^3^ Department of Pediatrics, Columbia University, New York, NY, United States; ^4^ LC Consulting LLC, Joliet, IL, United States; ^5^ Nutrition Science Communications, Nestlé USA, Inc., Gerber Products Company, Arlington, VA, United States; ^6^ Product Safety and Quality, Nestlé Product Technology Center, Konolfingen, Switzerland; ^7^ Scientific Affairs, Medical Scientific Regulatory Unit, Société des Produits Nestlé SA, Vevey, Switzerland

**Keywords:** guidance, families, nutrition, feeding practices, early childhood, lifelong health, healthy eating, infants and young children

## Abstract

Feeding infants and young children (IYC) is an emotional commitment for caregivers as they strive to achieve a sense of mastery, goodness, and accomplishment in providing nourishment for their children. Feeding practices are important during early childhood for lifelong health and well-being as behaviors related to healthy eating are established, and there is an interrelation between growth, gross/fine motor skills, and social-emotional behavior in shaping “learning to eat” for IYC. Mealtimes and feedings are opportunities for engagement with the family, formation of healthy habits, exposure to different foods, practicing eating skills, and learning to appreciate foods. The caregivers' role is pertinent as they influence and teach children “what” and “how” to eat and play a crucial role in supporting children's social, emotional, and cognitive development in relation to food and mealtimes. This mini review provides practical guidance for caregivers as their IYC “learn to eat.” Caregiver behaviors have changed, particularly in the choice of feeding methods, requiring an update on complementary feeding advice. Healthcare providers can encourage positive feeding practices. Family mealtimes provide opportunities for (1) bonding, (2) practicing and refining gross/fine motor, cognitive, language, and social-emotional skills, (3) offering a variety of nutrient-dense, appropriate textured foods, and (4) reinforcing the central role of the caregiver in establishing healthy eating patterns, a positive relationship with food, and joyful eating experiences. Healthcare providers play a pivotal role in raising awareness among caregivers about the importance of their role in feeding their child(ren).

## Introduction

**Figure 1:**
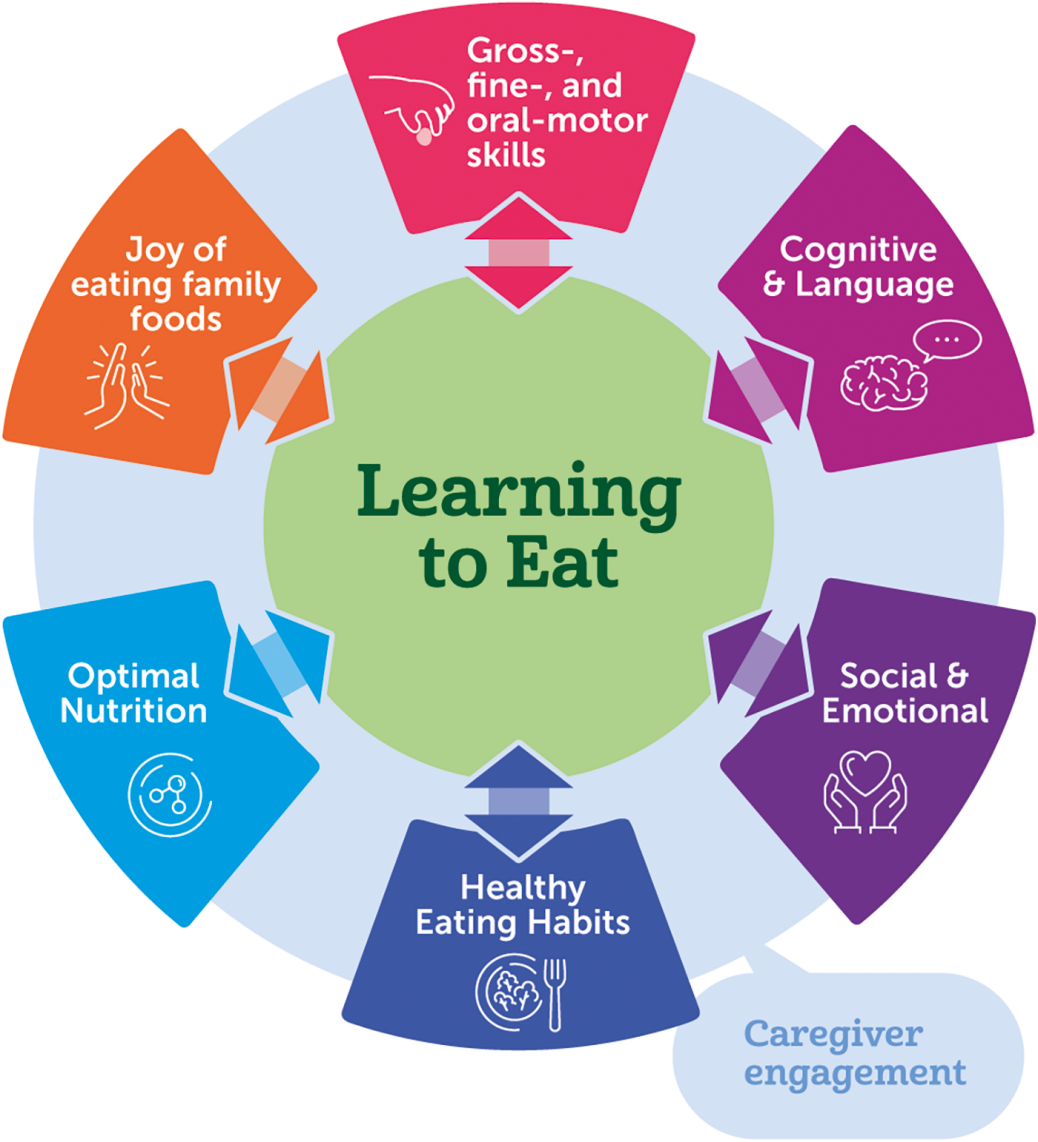
Learning to eat provides opportunities to build children's feeding skills and to nourish children's overall development through moments of family engagement and caregiver-child interactions.

The period of early childhood is crucial for development and lays the foundation for lifelong health and well-being (1–3). Nutrition plays a vital role in shaping developmental outcomes, and research suggests that there is a critical period when behaviors related to healthy eating are established (4, 5). The interrelation between growth, motor skills, and social-emotional behavior forms an axis where nutrition and feeding practices influence eating skills and shape “learning to eat” (Figure 1). Complementary feeding is defined as the period when pureed and solid foods are introduced around 6 months of age to support growth, development, and nutrition while promoting family engagement and form lifelong, healthy eating habits and practices (6, 7). While the focus of complementary feeding is often the immediate excitement, trepidation, and stepping stone of infants taking their first bite of food, the long-term goal of complementary feeding is to have enjoyable family mealtimes that support a healthy diet, support the social-emotional aspects of feeding, and follow hunger-satiety cues (7–13).

Eating integrates multiple skill domains (gross/fine motor, social-emotional, cognitive). Recommendations for complementary feeding traditionally focus on the types and amounts of foods that infants eat (what) and address responsive feeding (how) (14, 15). Mealtimes and feedings are occasions for exposure to different foods (tastes, textures), practicing eating (chewing, use of utensils), learning to appreciate foods, and exploration. The caregivers' role to provide healthy food to their infant cannot be overlooked. Interactive learning (process involving mutual responsiveness), the caregiver-child relationship and the caregiver's and child's readiness should be considered (16). Families influence and teach their children to eat a variety of foods, and these practices can be encouraged by healthcare providers (HCPs) through supportive guidance and education (17).

This paper reviews and summarizes current research and guidance updating evidence and guidance for caregivers as their IYC “learn to eat.” Caregiver behaviors have changed over time, particularly in choice of feeding methods where infant-led approaches [introducing solid foods to infants which gives control of the feeding process to the infant (18–22)] are becoming increasingly popular. An updated look at complementary feeding advice is warranted. Multiple aspects of feeding the growing infant are addressed and practical strategies for HCPs to encourage positive, joyful feeding practices are provided.

## Age of starting solids

Complementary feeding guidelines are generally consistent in recommending introducing complementary foods at about 6 months of age (3, 14, 23–25), not before 4 months of age nor delayed after 6 months of age (14, 23–25). As infants grow and become more active following the first 6 months of life, breastmilk alone no longer provides full nutritional requirements (3, 14, 26, 27); therefore, foods play a critical role in ensuring nutrient needs are met. Further, as infants advance in social-emotional development in concert with advancing fine motor skills, they exhibit curiosity and/or desire for flavors, textures, and foods they see their caregivers enjoy. Complementary feeding is a critical period in infant nutrition impacting outcomes later in life such as growth, non-communicable diseases, and food allergies (28).

## Developmental skills

All children develop at different rates; the ages outlined by the American Academy of Pediatrics (AAP) (29) and the US Centers for Disease Control (CDC) (30) at which certain skills are typically demonstrated are guides to use along with developmental readiness cues and determine when infants are ready to start solids. At 4–6 months, infants demonstrate emerging head control and loss of extrusor reflex (tongue thrust) (29). Sitting with support and wrapping fingers around objects placed in the hand like a spoon are gross/fine motor skills added to infants' repertoire at 4–6 months (4, 31). A spoon initially explored, with time, becomes a utensil for self-feeding. The oral cavity develops through 6 months where infants gain trunk stability and head control and sit independently (30, 32). Trunk stability is particularly important to support fine-motor and oral motor development and allow for self-feeding by infants. The prenatally developed feeding reflexes, including swallowing, sucking, gag, phasic bite, and rooting (33) gradually integrate with conscious actions until the development of mature eating. These reflexes integrate and disappear by the four-month period in the healthy infant (34). All these gross/fine motor milestones may indicate infants are ready to start complementary feeding. Acquisition of increasingly complex gross, fine, and oral motor skills is necessary for the developing child to progress through the journey of learning “how” to eat.

## Promoting healthy dietary patterns and texture exposure for children

Providing appropriate foods and beverages is critical for IYC to establish healthy dietary patterns. As defined by the US Dietary Guidelines for Americans 2020–2025 (DGA), “a healthy dietary pattern includes a variety of nutrient-dense fruits, vegetables, grains, protein foods (including lean meats, poultry, eggs, seafood, nuts, and seeds), dairy (including milk, yogurt, and cheese), and oils” (14). Expert guidelines not only recommend nutrient-dense foods, but also foods that are developmentally appropriate and diverse to ensure nutritional adequacy and support healthy eating habits (14, 35, 36). Limited, but consistent, evidence indicates that maternal dietary flavors in amniotic fluid and breastmilk promote early food acceptance in infants which are shaped by maternal dietary variety and responsive breastfeeding (37–40), while innate taste preferences direct infants toward sweet tastes (41, 42), making learning to eat vegetables and some fruits more challenging. However, repeated exposure can positively influence acceptance of novel tastes and textures (43–45). IYC have a heightened receptiveness to new foods, offering an early window of opportunity to foster dietary variety and positively influence a child's lifelong relationship with food (41, 46, 47).

Nutrient-dense complementary foods are central to the diet once solid foods are started. A diverse diet including foods from all food groups of various tastes and textures can ensure adequate nutrition and increase acceptance of foods. IYC develop their complex oral-motor skills needed for chewing nutrient dense foods and thereby increasing diet diversity. Purposeful introduction of the major food allergens [e.g., milk, eggs, shellfish, tree nuts, peanuts, wheat, and soy (48) and inclusive of fish and sesame in the US] should be early in the first year of life when other complementary foods are introduced in the diet (14, 23, 49) and often, to reduce the risk of development of food allergies (14, 49, 50). A healthy dietary pattern should be followed at every life stage incorporating nutrient dense foods, leaving little remaining energy for added sugars. Establishing these patterns can set a solid foundation for healthy eating and adequate nutrient intake.

Texture is an important consideration in IYC feeding. Food textures should be tailored to the developmental needs of the IYC and should change as the child gets older (24). The WHO recommends pureed, mashed, and semi-solid foods beginning at six months of age, finger foods that can be eaten alone by 8-month-old infants, and that most children should be eating the same types of foods consumed by the rest of the family by 12 months. It is crucial to ensure foods are appropriate in size and texture to minimize choking hazards. Moreover, some organizations specifically recommend introducing lumpy textures by 8–10 months, to decrease the risk of feeding difficulties, such as pickiness and food refusal (27, 51, 52).

Modern feeding trends focus on challenging infants (providing food types and textures that may require effort and ability to gum, chew, or eat and/or test oral-motor and gross-motor skills) and having infants self-feed various food types and textures. This is based on applying child development and learning approaches for 3–6-year-olds where alternatives to conventional education culture are explored and applied (53) to the infant feeding experience. An approach to consider is balancing guaranteed success with opportunities for exploration at infants' own pace such as offering practice foods where infants can step up towards new foods in terms of food texture. The typical progression is from pureed and smooth foods to soft textures with gradually increasing lumps to more complex textures and coarsely chopped foods with noticeable pieces. Using the carrot as an example, at about 6 months of age, it could be assumed an infant who has attained the appropriate developmental milestones would successfully be able to eat pureed carrots; to support the development of feeding skills, infants can be presented with carrots cut into appropriate shapes (stick, coin, half-moon, mashed) starting around 6 months of age and prepared to various suitable textures (i.e., soft cooked). This approach encourages exposure to and acceptance of more complex textures, promoting infants' abilities to handle different food forms as they grow.

## Exploring feeding methods and the importance of responsive and sensitive feeding

Several feeding methods are implemented by caregivers, like spoon-feeding, baby-led weaning, and baby-led feeding (28, 54–57). Spoon-feeding is a method where spoon-fed purees are given first, gradually increasing to more complex textures like mash to lumpy with a shift towards family foods with infants feeding themselves (54, 58). Baby-led weaning is an approach to introducing solid food based on infants' developmental readiness that allows infants to decide when to begin eating foods, what to eat, how quickly to eat, and how much to consume (21). Baby-led feeding combines spoon-feeding and baby-led weaning where purees may or may not be introduced at 4–6 months of age, with table foods, finger foods, mashes, lumpy foods, and smooth purees offered from 6 months on (56). This combination method, as it is sometimes referred to, is what most caregivers practice (59), rather than strictly following just one method. Pediatric associations and the DGA (14) do not recommend a specific feeding method to use, citing further research is needed. Feeding methods are an individualized choice; caregivers can introduce foods in the way that is most appropriate for their child's development, parenting style, and needs of the family.

Regardless of the method, responsive feeding is the recommended evidence-based approach for all feeding methods for IYC (9–11, 60–65). Responsive feeding encourages the child to eat autonomously and is characterized by caregiver recognition and response to the child's hunger and satiety cues. This involves reciprocal nurturing feeding practices between caregiver and child (54, 55, 58) and stresses the importance of acknowledging the bidirectional aspect of the feeding relationship. Responsive feeding supports IYC social-emotional development by meeting hunger and fullness cues in a sensitive and timely manner. This builds trust, security, food enjoyment in children (62), and a positive emotional connection between the child and caregiver. The benefits of responsive feeding are numerous and well-documented (60, 63, 66–69).

A recently coined feeding term is “sensitive feeding” (70). Sensitive feeding broadens the concept of responsive feeding to incorporate understanding and anticipating the child's point of view by sensitively responding to the child's signals to foster a pleasant and safe atmosphere during mealtimes (71). In turn, this facilitates the child's association of eating with positive emotions and encourages the willingness to eat and try new foods (70, 71). Respecting a child's experience during eating and avoiding intrusiveness during mealtimes is an important pillar in supporting an IYC in learning to eat.

## Creating a positive and nurturing feeding environment for child development

Without question, complementary feeding must provide optimal nutrition for the gross, fine, and oral motor development and growth of the IYC. Current evidence shows that the social-emotional, cognitive, and language-building aspects of feeding are also important and can be established and nurtured from the first bite (12, 72–74). Effective and stimulating communication exchange with caregivers and family members helps children express their preferences, ask for more food, or indicate when they are full. Fostering an engaging mealtime environment (positive affect of caregivers, conversing with those at the table, limiting distractions, honoring the role of both the child and the caregiver) can support better nutrition, better social-emotional outcomes, and promote the joy of eating among family members.

The AAP acknowledges the pivotal impact of early adult-child interactions in early brain development and recommends providing a rich and responsive language environment (75). One strategy that caregivers can implement is the 3Ts— “tune in, talk more, and take turns” (76). Family mealtimes provide a rich opportunity to practice this strategy; creating an environment with limited distractions encourages the caregiver to tune in to their child. During mealtime preparation, caregivers can talk to their IYC about what they're preparing, introducing numbers, colors (i.e., “I'm mashing up 3 pieces of a yellow banana”), increasing communication between the child and caregiver that can carry through mealtime. Taking turns inherently can occur during feeding, particularly as the IYC gets older. Early talk and interaction during specific periods in a child's early development where the brain is particularly receptive to learning certain skills, like language, and where experiences during that time can have a significant and lasting impact on future development can have positive effects on later language and cognitive development (77, 78). Feeding occasions provide caregivers with blocks where they can spend quality time with their IYC and simultaneously practice speaking and nurture communication.

Creating a positive and nurturing feeding environment to support social-emotional development is beneficial for a child's overall feeding and development. The connection between social-emotional development and responsive feeding demonstrates that feeding is not solely a physiological act, but a shared social-emotional experience. Self-regulation, attachment, social skills, food acceptance, autonomy, and emotional well-being all play a role in the development of feeding skills and the establishment of healthy eating habits (79, 80). In turn, a positive feeding environment offers opportunities for children to practice self-regulation, social skills, autonomy, and emotional well-being (12, 61). When caregivers respond sensitively to hunger, fullness, and other IYC cues, trust and a secure attachment between the IYC and caregiver are built, supporting social-emotional development (61, 81).

## Role of the caregiver and family

The role of the caregiver and family in learning to eat cannot be minimized. It is the responsibility of the caregiver to set the stage for sensitive feeding practices and responsive feeding. Maternal sensitive feeding behavior and positive affect, (smiling and complimenting rather than irritation or harshness) were positively associated with IYC vegetable intake and liking (70), demonstrating how the role of the caregiver can greatly influence the feeding experience of IYC. Eating, and role modeling how to eat, is associated with greater acceptance of novel foods (82). There is an interactive learning journey for accepting a variety of foods, and caregivers play a critical part in this journey. The dynamic between the caregiver and the IYC is bidirectional during the learning to eat journey; both caregiver and IYC need to be engaged and responsive to each other's cues. Creating an environment that is ideal for IYC feeding includes turning off screens and eating at the table where this dynamic can thrive (17, 83, 84).

## Practical applications and call to action

Caregivers are inundated with well-intentioned feeding advice, whether from social media, friends, or family members. However, much of this advice centers on what foods to offer and glossing over the caregiver role and holistic feeding guidance to choose what works best for their family within a science-based framework. For example, historically it has been advised to introduce a single food at a time, waiting days before introducing another (85–87); this advice is not based on science and does not afford adequate time to encourage a diverse diet with various foods and textures in infancy (85). It is the role of the HCP to educate the caregiver on current advice regarding the importance of a diversified diet (in flavors, textures, types of foods) including foods that are common allergens, an appropriate feeding environment, repeated exposure, responsive and sensitive feeding practices, and food safety (17).

**Figure 2:**
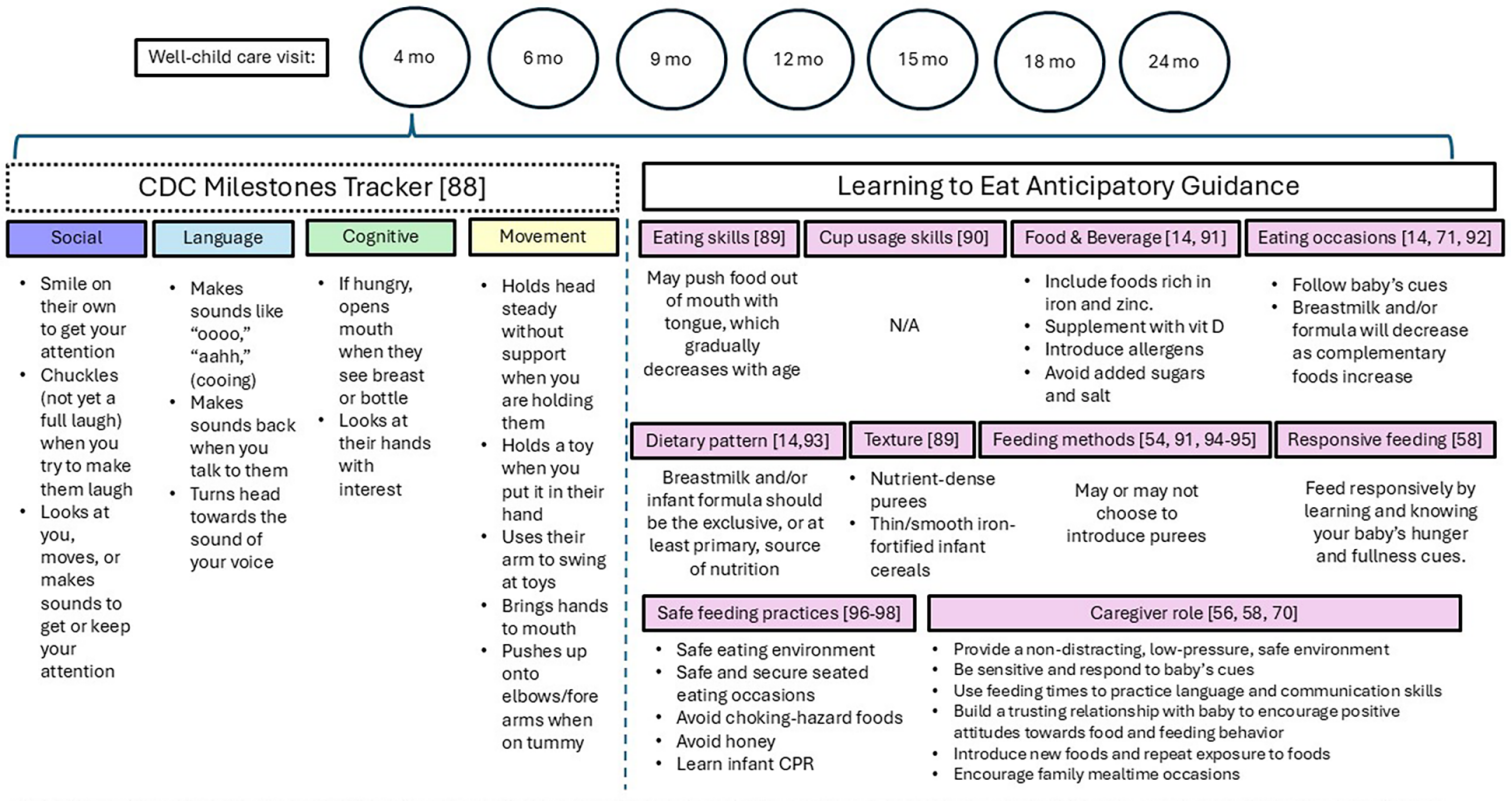
Overview of anticipatory developmental feeding guidance aligned with the 4-month well-child care visit. The above are not isolated events but build upon each other over time in an additive manner. As children grow and progress in age, they continue to develop and refine their skills, creating a foundation for further learning and development.

Dissemination of the message that caregivers are one of the most important “ingredients” in the feeding journey of their IYC can begin with the HCP. The 4-month well-child care visit (Figure 2) is an opportune time to discuss feeding goals and the caregiver's role. Some families mistakenly start complementary feeding prior to 4 months of age, so these conversations need to begin early, ideally at the 2 months visit as anticipatory guidance, and often, to reinforce the importance of starting solids around 6 months of age and not before 4 months of age. Open-ended questions can help assess the caregiver's understanding and aspiration for their IYC's feeding journey (Supplementary Figures S1–S6). This is a great way to set the stage for what the caregivers would like to accomplish (i.e., independent eaters) and their understanding of their role in the process. HCPs can educate families about “learning to eat”, focusing on developmental signs, the caregiver's readiness to move away from breast- or bottle-feeding, appropriate foods, the mealtime environment, and the caregiver's role through responsive and sensitive feeding, language, and engagement. Learning to eat is a journey that is greatly influenced by caregivers.

## Research gaps

Research gaps in early feeding practices of IYC need to be addressed to refine advice for caregivers. Feeding methods have received attention, but it remains unclear if one method has scientifically validated advantages. Studies with appropriate outcomes, follow-up, and comparison groups are needed to inform HCPs and caregivers. Effective feeding methods and strategies must be identified to ensure children receive proper nutrition. This includes understanding the impact on growth and development, including social/emotional, as well as barriers caregivers may face. Understanding cultural beliefs and practices related to feeding is crucial for effective caregiver education. Dietary practices vary across cultures, but a generalized knowledge base that can be tailored to each family is ideal. This knowledge is essential for developing caregiver-education programs that support children's healthy development. Overall, addressing research gaps is vital for evidence-based guidance and support to caregivers in feeding their children and promoting their development.

## Conclusion

Family mealtimes provide opportunities for bonding between the IYC and caregivers and for practicing and refining not only gross, fine, and oral motor skills, but cognitive, language, and social-emotional skills. Nutrient-dense foods and those that commonly cause allergies should be offered in textures, shapes, and sizes appropriate for the developmental stage of the IYC. The caregiver-child dynamic relationship is central to establishing healthy dietary patterns and joyful associations from the start. Regardless of feeding method used, responsive and sensitive feeding practices where the caregiver is attuned to the IYC feeding cues and an active role model for exploring foods are of utmost importance. The HCP is pivotal in bringing awareness to caregivers of just how crucial their role is in the feeding journey for their child that sets the tone for a life of healthy eating.
