# Supplementary material for: Essential elements for learning to eat: guidance to support families with infants and young children
Source: Front Pediatr. 2025 Mar 27;13:1493780. doi: 10.3389/fped.2025.1493780 (PMC11983468; doi:10.3389/fped.2025.1493780)
Supplement: Supplementary file 1 [file Presentation1.pdf]

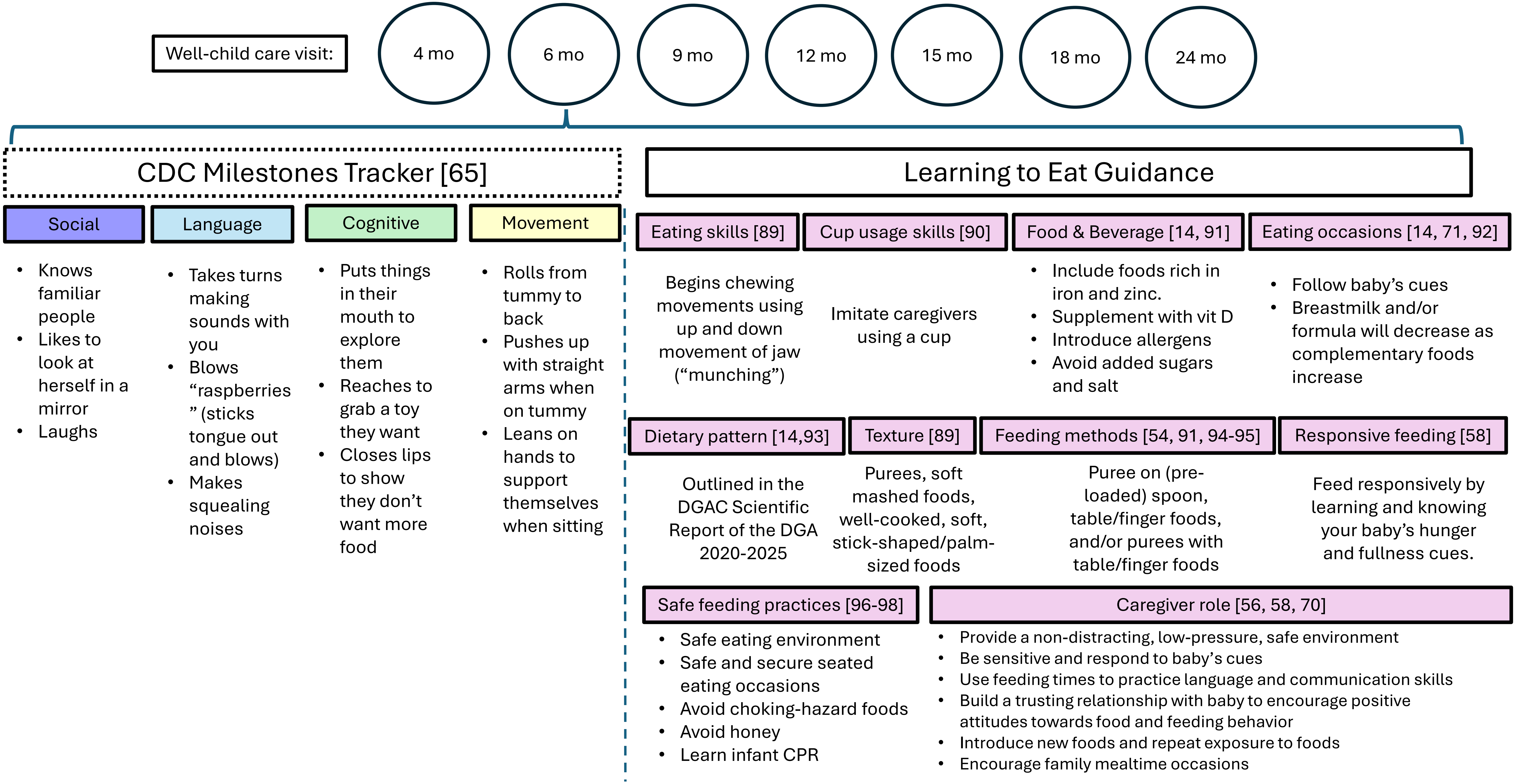

Supplementary Figure 1. Overview of developmental feeding guidance aligned with the 6-month well-child care visit. The below are not isolated events but build upon each other over time in an additive manner. As children grow and progress in age, they continue to develop and refine their skills, creating a foundation for further learning and development.

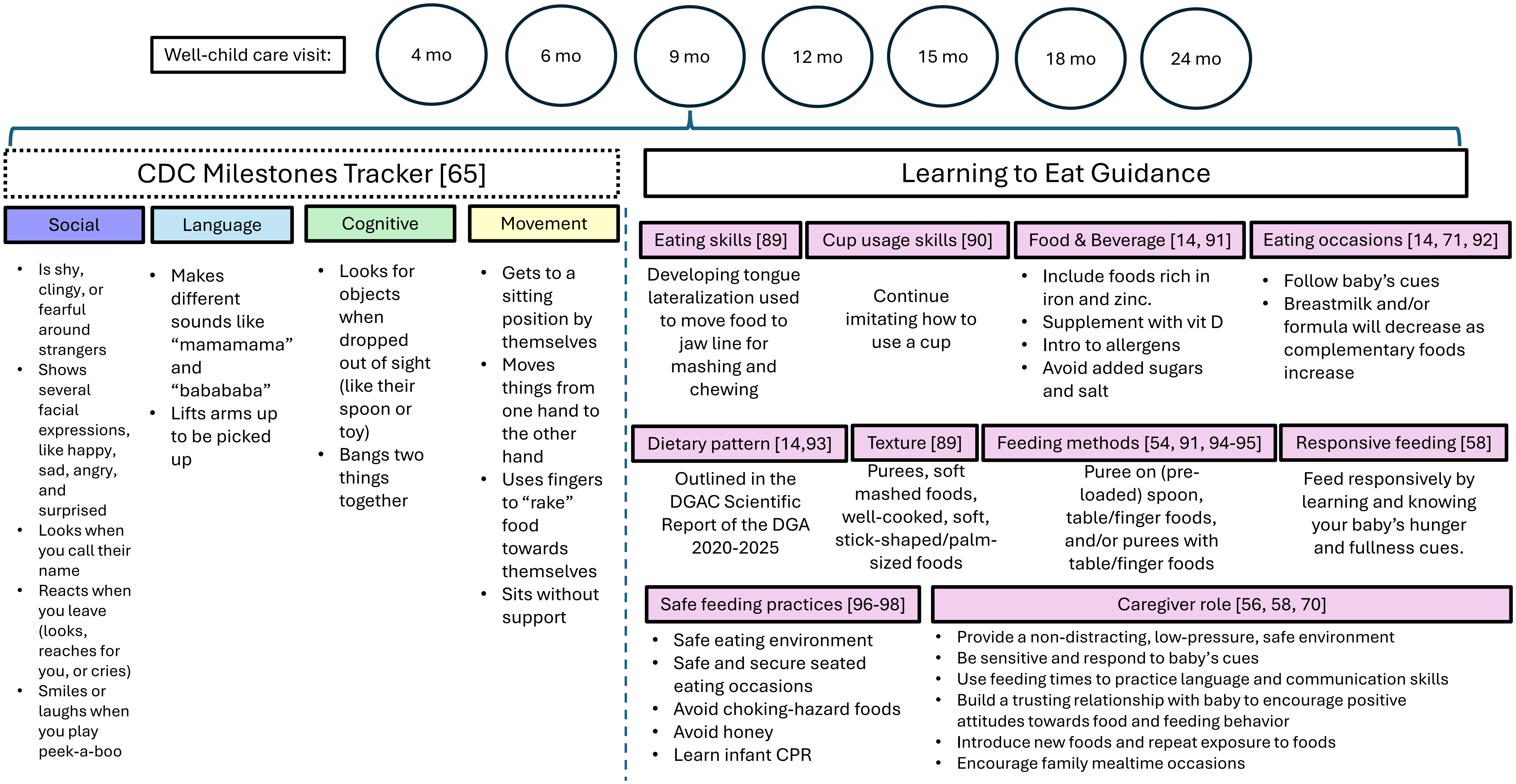

Supplementary Figure 2. Overview of developmental feeding guidance aligned with the 9-month well-child care visit. The below are not isolated events but build upon each other over time in an additive manner. As children grow and progress in age, they continue to develop and refine their skills, creating a foundation for further learning and development.

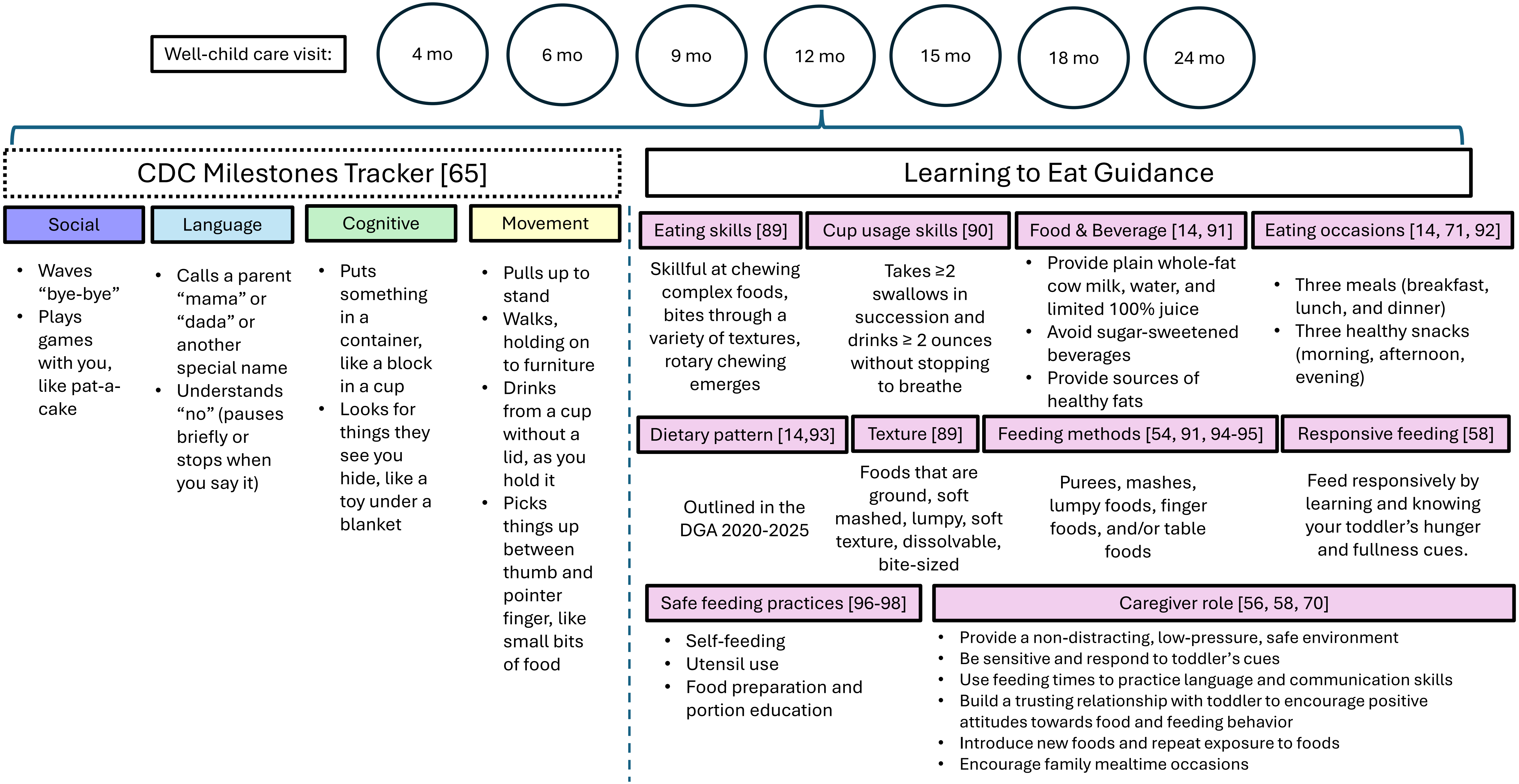

Supplementary Figure 3. Overview of developmental feeding guidance aligned with the 12-month well-child care visit. The below are not isolated events but build upon each other over time in an additive manner. As children grow and progress in age, they continue to develop and refine their skills, creating a foundation for further learning and development.

Well-child care visit: 4 mo | 6 mo | 9 mo | 12 mo | 15 mo | 18 mo | 24 mo

CDC Milestones Tracker [65]

Social
- Copies other children while playing, like taking toys out of a container when another child does
- Shows you an object they like
- Claps when excited
- Hugs stuffed doll or other toy
- Shows you affection (hugs, cuddles, or kisses you)

Language
- Tries to say one or two words besides "mama" or "dada," like "ba" for ball or "da" for dog
- Looks at familiar object when you name it
- Follows directions given with both a gesture and words. For example, they give you a toy when you hold out your hand and say, "Give me the toy."

Cognitive
- Tries to use things the right way, like a phone, cup, or book
- Stacks at least two small objects, like blocks

Movement
- Takes a few steps on their own
- Uses fingers to feed themselves some food

Learning to Eat Guidance

Eating skills [89]
Rotary chewing becomes more coordinated, may spit out harder to chew skins/flesh of fruits/vegetables

Cup usage skills [90]
Learning to orient the hand more precisely to the position of objects – hands around cup

Food & Beverage [14, 91]
- Provide plain whole-fat cow milk, water, and limited 100% juice
- Avoid sugar-sweetened beverages
- Provide sources of healthy fats

Eating occasions [14, 71, 92]
- Three meals (breakfast, lunch, and dinner)
- Three healthy snacks (morning, afternoon, evening)

Dietary pattern [14,93]
Outlined in the DGA 2020-2025

Texture [89]
Cut-up family/table foods, chunks/diced fruits/vegetables, shredded/ground meats

Feeding methods [54, 91, 94-95]
Self-feeding table foods and finger foods

Responsive feeding [58]
Feed responsively by learning and knowing your toddler's hunger and fullness cues.

Safe feeding practices [96-98]
- Supervised eating occasions
- Ingestion of foods vs. non-foods
- Consumption of foods vs. insertion of foods in nose or ears

Caregiver role [56, 58, 70]
- Provide a non-distracting, low-pressure, safe environment
- Be sensitive and respond to toddler's cues
- Use feeding times to practice language and communication skills
- Build a trusting relationship with toddler to encourage positive attitudes towards food and feeding behavior
- Introduce new foods and repeat exposure to foods
- Encourage family mealtime occasions

Supplementary Figure 4. Overview of developmental feeding guidance aligned with the 15-month well-child care visit. The below are not isolated events but build upon each other over time in an additive manner. As children grow and progress in age, they continue to develop and refine their skills, creating a foundation for further learning and development.

Well-child care visit: 4 mo | 6 mo | 9 mo | 12 mo | 15 mo | 18 mo | 24 mo

CDC Milestones Tracker [65]

Social
- Moves away from you, but looks to make sure you are close by
- Points to show you something interesting
- Puts hands out for you to wash them
- Looks at a few pages in a book with you
- Helps you dress them by pushing arm through sleeve or lifting up foot

Language
- Tries to say three or more words besides "mama" or "dada"
- Follows one-step directions without any gestures, like giving you the toy when you say, "Give it to me."

Cognitive
- Copies you doing chores, like sweeping with a broom
- Plays with toys in a simple way, like pushing a toy car

Movement
- Walks without holding on to anyone or anything
- Scribbles
- Drinks from a cup without a lid and may spill sometimes
- Feeds themselves with their fingers
- Tries to use a spoon
- Climbs on and off a couch or chair without help

Learning to Eat Guidance

Eating skills [89]
Regulation of jaw speed depending on food consistency

Cup usage skills [90]
Dynamic relationship between eye-hand-mouth coordination involved in independent cup usage

Food & Beverage [14, 91]
- Provide plain whole-fat cow milk, water, and limited 100% juice
- Avoid sugar-sweetened beverages
- Provide sources of healthy fats

Eating occasions [14, 71, 92]
- Three meals (breakfast, lunch, and dinner)
- Three healthy snacks (morning, afternoon, evening)

Dietary pattern [14,93]
Outlined in the DGA 2020-2025

Texture [89]
Cut-up family/table foods, chunks/diced fruits/vegetables, shredded/ground meats

Feeding methods [54, 91, 94-95]
Self-feeding table foods and finger foods

Responsive feeding [58]
Feed responsively by learning and knowing your toddler's hunger and fullness cues.

Safe feeding practices [96-98]
- Supervised eating occasions
- Ingestion of foods vs. non-foods
- Consumption of foods vs. insertion of foods in nose or ears

Caregiver role [56, 58, 70]
- Provide a non-distracting, low-pressure, safe environment
- Be sensitive and respond to toddler's cues
- Use feeding times to practice language and communication skills
- Build a trusting relationship with toddler to encourage positive attitudes towards food and feeding behavior
- Introduce new foods and repeat exposure to foods
- Encourage family mealtime occasions

Supplementary Figure 5. Overview of developmental feeding guidance aligned with the 18-month well-child care visit. The below are not isolated events but build upon each other over time in an additive manner. As children grow and progress in age, they continue to develop and refine their skills, creating a foundation for further learning and development.

Well-child care visit: 4 mo | 6 mo | 9 mo | 12 mo | 15 mo | 18 mo | 24 mo

CDC Milestones Tracker [65]

Social
- Notices when others are hurt or upset, like pausing or looking sad when someone is crying
- Looks at your face to see how to react in a new situation

Language
- Points to things in a book when you ask, like "Where is the bear?"
- Says at least two words together, like "More milk."
- Points to at least two body parts when you ask them to show you.
- Uses more gestures than just waving and pointing, like blowing a kiss or nodding yes

Cognitive
- Holds something in one hand while using the other hand; for example, holding a container and taking the lid off
- Tries to use switches, knobs, or buttons on a toy
- Plays with more than one toy at the same time, like putting toy food on a toy plate

Movement
- Kicks a ball
- Runs
- Walks (not climbs) up a few stairs with or without help
- Eats with a spoon

Learning to Eat Guidance

Eating skills [89]
Continues to refine chewing movements (i.e., reduced duration and number of chewing cycles)

Cup usage skills [90]
Child can release cup into caregivers' hand or onto a surface, will be clumsy at first

Food & Beverage [14, 91]
- Transition to plain low-fat cow milk, water, and limited 100% juice
- Avoid sugar-sweetened beverages
- Provide sources of healthy fats

Eating occasions [14, 71, 92]
- Three meals (breakfast, lunch, and dinner)
- Three healthy snacks (morning, afternoon, evening)

Dietary pattern [14,93]
Outlined in the DGA 2020-2025

Texture [89]
Crispy, chewy, creamy, dissolvable, hard, juicy, leafy, lumpy, pureed, rubbery, slippery, soft, sticky

Feeding methods [54, 91, 94-95]
Self-feeding table foods and finger foods

Responsive feeding [58]
Feed responsively by learning and knowing your preschooler's hunger and fullness cues.

Safe feeding practices [96-98]
- Supervised eating occasions
- Ingestion of foods vs. non-foods
- Consumption of foods vs. insertion of foods in nose or ears

Caregiver role [56, 58, 70]
- Provide a non-distracting, low-pressure, safe environment
- Be sensitive and respond to preschooler's cues
- Use feeding times to practice language and communication skills
- Build a trusting relationship with preschooler to encourage positive attitudes towards food and feeding behavior
- Introduce new foods and repeat exposure to foods
- Encourage family mealtime occasions

Supplementary Figure 6. Overview of developmental feeding guidance aligned with the 24-month well-child care visit. The below are not isolated events but build upon each other over time in an additive manner. As children grow and progress in age, they continue to develop and refine their skills, creating a foundation for further learning and development.
